# A healthy HLA-matched baby born by using a combination of aCGH and Karyomapping: the first latin american case

**DOI:** 10.5935/1518-0557.20170063

**Published:** 2017

**Authors:** Andrea Delgado, Guillermo Llerena, Rosmary Lopez, Jimmy Portella, Naomi Inoue, Luis Noriega-Hoces, Luis Guzman

**Affiliations:** 1 PRANOR Laboratorio. Grupo de Reproducción Asistida. San Isidro. Lima. Peru.; 2 Clinica Concebir. Calle Los Olivos 364. San Isidro. Lima 31. Peru.; 3 Reprogenetics Latinoamérica, Lima-Peru.

**Keywords:** HLA matching, aCGH, karyomapping

## Abstract

PGD for HLA typing is a procedure that can be performed when an affected child requires a transplant to treat a non-hereditary disorder related to the hematopoietic and/or immune system. Hematopoietic stem cell transplantation from an HLA-identical donor provides the best treatment option. Three conventional ovarian stimulation procedures for IVF were performed in a couple with a 10-year-old child diagnosed with T-cell acute lymphoblastic leukemia of high risk. Trophectoderm biopsy and aCGH examination were performed on 15 blastocysts, three on the first IVF procedure, four on the second cycle, and eight on the third. Three euploid blastocysts HLA-compatible with the genome of the affected child were identified. One euploid blastocyst HLA-compatible with the affected child was warmed and transferred, resulting in an HLA-matched live birth. In conclusion, combined aCGH for aneuploidy screening and Karyomapping may be performed in a single biopsy procedure.

## INTRODUCTION

T-cell acute lymphoblastic leukemia is an immature lymphoid tumor characterized by diffuse infiltration of the bone marrow by malignant hematopoietic cells expressing immature T-cell markers. Clinically, patients with T-cell acute lymphoblastic leukemia have elevated white blood cell counts and hematopoietic insufficiency, neutropenia, anemia, and thrombocytopenia. In addition, they often show thymic mediastinal masses and meningeal infiltration of the central nervous system at the time of diagnosis. Immature T-cell tumors presenting as thymic masses with limited bone marrow infiltration are diagnosed instead as T-lymphoblastic lymphomas (Belver & Ferrando, 2016).

Preimplantation genetic diagnosis (PGD) has been applied as a selection tool to avoid inheritable diseases (Ren *et al*., 2016). However, the primary limitation of PGD for single gene disorders is the small amount of DNA. Whole genome amplification is required to detect the mutation. DNA amplification may produce a preferential amplification of one parental allele, which can cause heterozygous loci to appear homozygous. This process, known as allele dropout (ADO), may lead to misdiagnosis. In addition, the extreme sensitivity of amplification and detection methods makes them more susceptible to contamination. To avoid these potential causes of misdiagnosis, standard practice includes targeted haplotyping of the genetic locus by multiplex amplification of one or more closely related or intragenic polymorphic markers with or without direct detection of mutations (Natesan *et al.*, 2014).

With Karyomapping, the entire parental genome can be haplotyped using the high-density single nucleotide polymorphism (SNP) method. Karyomapping provides an integral diagnostic method based on the ligation of single-gene defects. Genotyping parents and close relatives with known diseases eliminates the need for custom test development, and since Karyomapping defines four sets of SNP markers for each of the parental chromosomes, it allows the simultaneous execution of molecular cytogenetic analysis (Thornhill *et al*., 2015).

PGD for HLA typing is a procedure that can be performed as a single indication when the affected child requires a transplant to treat a non-hereditary disorder related to the hematopoietic and/or immune system, such as certain types of leukemia, or simultaneously with PGD to rule out a disease linked to a single family gene (E.g. β thalassemia) and allow pregnancy to develop with an unaffected HLA-compatible embryo (Kakourou *et al*., 2016).

Hematopoietic stem cell transplantation from an HLA-identical donor is the best treatment option for bone marrow transplantion, as it reduces the incidence of graft-versus-host disease (Soni *et al*., 2014). This case report describes what was, to our knowledge, the first PGD live birth of an HLA-matched child in Latin America using aCGH for chromosome screening and Karyomapping for HLA matching.

## CASE REPORT

In November of 2014, a couple came to our assisted reproduction clinic with plans of having a child. They had been living together for 15 years and had two living children, a boy and girl. Recently, however, the couple had a miscarriage (2014).

Their 10-year-old boy had been diagnosed with T-cell acute lymphoblastic leukemia, but tests for bone marrow transplantation performed at the National Institute for Neoplastic Diseases in Peru revealed he and his sister were not histocompatible. After having searched the Bone Marrow Donors Worldwide database, they were offered the possibility of undergoing IVF with PGD. They were informed of the scope and limitations of PGD in HLA typing.

Following our clinical protocol, the 36-year-old woman was instructed to perform a hormone profile test and undergo pelvic ultrasound examination. The tests revealed she had polycystic ovaries, and treatment with Metformin was prescribed (Metformin - 1500 mg/day). Though her hormone profile was normal, prolactin was found to be elevated (39 ng/mL) and she was treated with Cabergoline (Dostinex - Pfizer).

The 42-year-old man was instructed to undergo conventional sperm analysis. According to the WHO 2010 manual (World Health Organization, 2010), he was normozoospermic. The couple was also advised to undergo psychological counseling. Considering their clinical history and the evident determination of the couple, it was decided they would undergo IVF cycles in combination with PGD for HLA matching.

### IVF protocol

Three controlled ovarian stimulation (COS) cycles were performed. The patients were properly advised about the procedures and signed informed consent terms.

The first COS cycle was performed in December of 2014. Ovarian induction was done with 150 IU of FSH/LH (Bravelle - Ferring) and 150 IU of hp-HMG (Menopur-Ferring) from day 2 to day 10 of the cycle. Ant-GnRH (Orgalutran-Merck) with follicle ≥14 (d 8, 9, and 10), and ovulation time was synchronized with r-hCG (Ovidrel-Merck). After oocyte retrieval, 34 oocytes were obtained; 17 were mature, eight were fertilized and three blastocysts were vitrified for banking. Vitrification was performed according to cryotech instructions (Kuwayama, 2007)

The second COS cycle was performed in February of 2015. Follicular growth was induced with 150 IU of FSH/LH (Bravelle-Ferring) and 75 IU of hp-HMG (Menopur-Ferring) from day 3 to day 10 of the cycle. Ant-GnRH (Orgalutran-Merck) with follicle ≥14 (d 8, 9, and 10) and ovulation was triggered with Ana-GnRH (Gonapeptil-Ferring). Twenty-eight oocytes were aspirated; 16 were mature, 10 were fertilized and four blastocysts were vitrified.

The last COS cycle was performed in April of 2015. Patient was administrated 150 IU of FSH/LH (Bravelle - Ferring) and 75 IU of hp-HMG (Menopur-Ferring) from day 2 to day 11 of the cycle. Ant-GnRH (Cetrotide-Merck Serono) with follicle ≥14 (d 8, 9, 10, and 11) and ovulation was synchronized with Ana-GnRH (Gonapeptil-Ferring). In this cycle, 33 oocytes were aspirated; 26 were mature, 18 were fertilized and eight blastocysts were biopsied on days 5/6. On the same day, all blastocyst generated in previous cycles were warmed and biopsied. All blastocysts were vitrified for genetics analysis. (Table 1).

**Table 1:** A summary of the clinical IVF cycle outcome.

N° of cycles	I	II	III	Overall
N° oocytes retrieved	34	28	33	95
N° of mature oocytes (%)	17 (50%)	16 (57%)	26 (79%)	59 (62%)
N° of zygotes	8	10	18	36
% of oocyte fertilized	47.05 %	62.5 %	69.23 %	61.02 %
N° of biopsied blastocyst	3	4	8	15
% of embryos analyzed by aCGH	37.5 %	40 %	44.44 %	41.65 %
Number of euploid blastocyst (%)	3 (100%)	1 (25%)	4 (50%)	8 (53%)
N° of HLA-matched embryos	0	0	3	3 (20%)

Trophectoderm biopsy and aCGH examination were performed on 15 blastocysts, three on the first IVF procedure, four on the second cycle, and eight on the third. PGS with aCGH results showed that eight blastocysts were euploid (Table 2).

**Table 2:** aCGH results from all analyzed blastocysts.

Emb	XY	1	2	3	4	5	5	7	3	9	10	11	12	13	14	15	15	17	13	19	20	21	22	Interpretation
1	XY	2	2	2	2	2	2	2	2	2	2	2	2	2	2	2	2	2	2	1	2	2	2	ABNORMAL, MONOSOMY 19
**3**	**XY**	**2**	**2**	**2**	**2**	**2**	**2**	**2**	**2**	**2**	**2**	**2**	**2**	**2**	**2**	**2**	**2**	**2**	**2**	**2**	**2**	**2**	**2**	**NORMAL**
**5**	**XY**	**2**	**2**	**2**	**2**	**2**	**2**	**2**	**2**	**2**	**2**	**2**	**2**	**2**	**2**	**2**	**2**	**2**	**2**	**2**	**2**	**2**	**2**	**NORMAL**
10	XY	2	2	2	2	2	2	2	2	2	2	2	2	2	3	2	3	2	2	2	2	1	2	ABNORMAL COMPLEX
11	XY	2	2	2	2	2	2	2	2	2	2	2	2	2	2	2	2	3	2	2	2	2	2	ABNORMAL, TRISOMY 17
**12**	**XY**	**2**	**2**	**2**	**2**	**2**	**2**	**2**	**2**	**2**	**2**	**2**	**2**	**2**	**2**	**2**	**2**	2	**2**	**2**	**2**	**2**	**2**	**NORMAL**
**14**	**XY**	**2**	**2**	**2**	**2**	**2**	**2**	**2**	**2**	**2**	**2**	**2**	**2**	**2**	**2**	**2**	**2**	**2**	**2**	**2**	**2**	**2**	**2**	**NORMAL**
16	XX	2	2	2	2	2	2	2	2	2	2	2	2	2	2	2	3	2	2	2	2	2	2	ABNORMAL, TRISOMY 16
**19**	**XX**	**2**	**2**	**2**	**2**	**2**	**2**	**2**	**2**	**2**	**2**	**2**	**2**	**2**	**2**	**2**	**2**	**2**	**2**	**2**	**2**	**2**	**2**	**NORMAL**
**20**	**XY**	**2**	**2**	**2**	**2**	**2**	**2**	**2**	**2**	**2**	**2**	**2**	**2**	**2**	**2**	**2**	**2**	**2**	**2**	**2**	**2**	**2**	**2**	**NORMAL**
**21**	**XY**	**2**	**2**	**2**	**2**	**2**	**2**	**2**	**2**	**2**	**2**	**2**	**2**	**2**	**2**	**2**	**2**	**2**	**2**	**2**	**2**	**2**	**2**	**NORMAL**
22	XX	2	2	2	2	2	2	2	2	2	2	2	2	2	2	2	2	2	2	2	2	2	3	ABNORMAL, TRISOMY 22
23	XX	2	2	2	2	2	2	2	2	2	2	2	2	2	2	2	2	2	2	2	2	2	3	ABNORMAL, TRISOMY 22
24	XY	2	2	2	2	2	2	2	2	2	3	2	2	2	2	2	2	2	2	2	3	2	2	ABNORMAL, TRISOMY 10 AND 20
**25**	**XX**	**2**	**2**	**2**	**2**	**2**	**2**	**2**	**2**	**2**	**2**	**2**	**2**	**2**	**2**	**2**	**2**	**2**	**2**	**2**	**2**	**2**	**2**	**NORMAL**

The same DNA isolated and amplified for aCGH was used to perform PGD with Karyomapping. In this case, the HLA region has 128 informative SNPs in the mother and 78 in the father. SNPs were classified as compatible or non-compatible with respect to the analysis of the same SNPs in the affected child's cells.

The SNPs reported as compatible were grouped as P1 (paternal origin) and M1 (maternal origin) (Table 3). In order for an embryo to be diagnosed as compatible, it had to present the M1/P1 pattern (Table 3). Three euploid blastocysts HLA-compatible with the affected child genome were identified (Table 4).

**Table 3:** Individuals reported as compatible in Karyomapping were grouped as P1 (paternal origin) and M1 (maternal origin).

HLA - RESULTS		INTERPRETATION
**Male**	Father	N/A
Predicted phase	P1/****P2****	
**Female**	Mother	N/A
Predicted phase	Ml/****M2****	
**Reference: Son**	Son	N/A
Predicted phase	Ml/Pl	

HLA Key:

M1 represents SNPs supporting MATERNAL COMPATIBLE PHASE

P1 represents SNPs supporting PATERNAL COMPATIBLE PHASE

**M1** in **bold** represents SNPs supporting **MATERNAL NON-COMPATIBLE PHASE**

**P1** in **bold** represents SNPs supporting **PATERNAL NON-COMPATIBLE PHASE**

**Table 4:** HLA-matching results for all euploid embryos. This information includes the predictive phase, in which solely the M1/P1 pattern is considered for purposes of interpreting embryo compatibility.

HLA-RESULTS		INTERPRETATION
**Embryo**	**3**	**COMPATIBLE**
Predicted phase	M1, P1	
Supporting Evidence	53 key SNP5 support M1	
	23 key SNPs support P1	
**Embryo**	**5**	**COMPATIBLE**
Predicted phase	M1, P1	
Supporting Evidence	72 key SNPs support M1	
	25 key SNFs support P1	
**Embryo**	12	NON-COMPATIBLE
Predicted phase	**M2**, P1	
Supporting Evidence	55 key SNPs support **M2**	
	30 key SNFs support P1	
**Embryo**	14	**COMPATIBLE**
Predicted phase	M1, P1	
Supporting Evidence	63 key SNPS support Ml	
	21 key SNFS support P1	
**Embryo**	19	NON-COMPATIBLE
Predicted phase	**M2, P2**	
Supporting Evidence	54 key SNPs support **M2**	
	41 key SNFs support **P2**	
**Embryo**	20	NON-COMPATIBLE
Predicted phase	**M2**, P1	
Supporting Evidence	53 key SNPs support **M2**	
	24 key SNPs support P1	
**Embryo**	21	NON-COMPATIBLE
Predicted phase	**M2, P2**	
Supporting Evidence	47 key SNPs support **M2**	
	37 key SNPs support **P2**	
**Embryo**	25	NON-COMPATIBLE
Predicted phase	**M2**, P1	
Supporting Evidence	55 key SNPs support **M2**	
	25 key SNPs support P1	

### Endometrium preparation for Embryo Transfer

In the middle of the luteal phase (day 21) of the previous menstrual cycle the patient was administered Leuprolide Acetate (Lupron Depot^®^ - Abbott), a GnRH agonist, 3.75 mg intra muscular. On the first day of subsequent menses, transvaginal ultrasound was performed and hormone therapy with Estradiol Valerate (Progynova^®^ - Shering) was initiated in a variable dose protocol, with an oral dose of 4 mg/day from day 1 to day 7, and 6 mg/day from day 8 until an endometrial thickness of 10 mm and trilaminar pattern was reached, on day 18. At that time, Micronized Progesterone 600 mg vaginal per day was initiated and the dose of Estradiol Valerate was increased to 8 mg/day.

Deferred embryo transfer was performed in July of 2015. After 5.5 days of progesterone, a single HLA-matched embryo was warmed and cultured. Two hours later, the embryo re-expanded and was transferred with the aid of ultrasound guidance. Two weeks after embryo transfer, a first β-HCG score of 4,420 mIU/ml was obtained; and two days later, the value was 10,494 mIU/ml.

 The patient went regularly to her prenatal checkups at Clínica Concebir San Isidro - Lima - Peru, and on March 28^th^, 2016, a cesarean section was performed, resulting in the birth of a male newborn weighing 3.350 kg with an Apgar score of 9/9. At birth, umbilical cord stem cells were collected and cryopreserved.

At the time this report was being written, the parents performed a first post-natal HLA-compatibility test and found that both children had matching HLA, which made it possible for bone marrow transplantation to occur.

## DISCUSSION

Fertility treatments may be combined with genetic tests to prevent the occurrence of chromosomal alterations (aCGH) or genetic diseases (Karyomapping). Verlinsky *et al*. (2001) described the first case of PGD + HLA-typing for Fanconi anemia. To our knowledge, the present case report describes the first live birth of an HLA-matched baby tested with PGD in Latin America by using a combination of aCGH and Karyomapping.

This case report described two clinical challenges. The first was the PCO-like ovaries. An antagonist GnRH protocol was used and modified after the first cycle, because of the low quality of oocytes determined by low blastocyst formation. In the following cycles the dose of hp-HMG was decreased on account of the polycystic ovaries. A GnRH agonist was also used as a final trigger for oocyte maturation to minimize the risk of ovarian hyperstimulation syndrome (Kol, 2004). In women with polycystic ovary syndrome or polycystic ovaries, Metformin before or during assisted reproductive technology cycles increases clinical pregnancy rates and decreases the risk of ovarian hyperstimulation syndrome. However, there is no conclusive evidence of benefits in life birth rates (Tso *et al*., 2015).

The second challenge was the combination of PGD techniques (aCGH and Karyomapping). There are few reports describing this type of protocol. One of the studies was performed between 2007 and 2014 and enrolled 12 couples interested in taking part in a PGD-HLA-typing program. All couples had children affected by either a genetic or an acquired disease affecting their hematopoietic and/or immune system. Seven couples were treated in 26 cycles and two healthy HLA-matched babies were born, leading to a live birth rate of 28.6% per transfer and 7.7% per initiated cycle (Fernández *et al*., 2014). Nevertheless, PGD was not combined with chromosome screening for aneuploidies.

Three IVF cycles were performed, since a large number of embryos was required to obtain a chromosomally euploid HLA-matched embryo. The multidisciplinary team, which included gynecologists, biologists and geneticists, considered the conditions of these types of diagnoses were very demanding. In the three IVF cycles performed, trophectoderm biopsies were performed on 15 blastocysts, of which eight (53.3%) were euploid on aCGH; and three (20%) were HLA-compatible with the affected child. Similar results have been reported in a French study, in which 19% (3 out of 16) of the embryos were unaffected and HLA-compatible (Lamazou *et al*., 2011).

IVF combined with HLA-typing has become another option to find HLA-matched donors. The literature indicates that the chances of finding an HLA-identical donor within a family or in Hematopoietic stem cell banks are low (Fernández *et al*., 2014). In pathological conditions, PGD can be used to select unaffected HLA-matched embryos for transfer. After birth, umbilical cord and blood stem cells may be collected for transplantation into an affected child, a proven strategy to cure these children (Natesan *et al*., 2014).

Hematopoietic stem cell (HSC) transplantation from an HLA-identical donor is the best therapeutic option for genetic diseases affecting the hematopoietic and/or immune system in children, and may be an option for individuals with acquired diseases as well (Gaziev *et al*., 2000; Hellani *et al*., 2012).

Regarding legislation, there is no regulation for ART procedures in Peru. All clinical and laboratory practices were performed following the highest ethical standards and in close compliance with European legislation (Van de Velde *et al*., 2009). Nevertheless, there are ethical concerns when this type of procedure is carried out (Pennings *et al*., 2002).

In conclusion, combined aCGH for aneuploidy screening and Karyomapping was performed in a single biopsy procedure, resulting in an HLA-matched live birth. This type of procedure may be extended to other single gene disorders.
